# Redox determines greenhouse gas production kinetics and metabolic traits in water-saturated thawing permafrost peat

**DOI:** 10.1093/ismeco/ycaf009

**Published:** 2025-03-03

**Authors:** Eira Catharine Lødrup Carlsen, Jing Wei, Franck Lejzerowicz, Sigrid Trier Kjær, Sebastian Westermann, Dag O Hessen, Peter Dörsch, Alexander Eiler

**Affiliations:** Section for Aquatic Biology and Toxicology, Department of Biosciences, University of Oslo, 0371 Oslo, Norway; Center for Biogeochemistry in the Anthropocene, University of Oslo, 0371 Oslo, Norway; Section for Aquatic Biology and Toxicology, Department of Biosciences, University of Oslo, 0371 Oslo, Norway; Center for Biogeochemistry in the Anthropocene, University of Oslo, 0371 Oslo, Norway; Section for Aquatic Biology and Toxicology, Department of Biosciences, University of Oslo, 0371 Oslo, Norway; Center for Biogeochemistry in the Anthropocene, University of Oslo, 0371 Oslo, Norway; Faculty of Environmental Sciences and Natural Resource Management, Norwegian University of Life Sciences, NMBU, 1432 Ås, Norway; Center for Biogeochemistry in the Anthropocene, University of Oslo, 0371 Oslo, Norway; Department of Geosciences, University of Oslo, 0371 Oslo, Norway; Section for Aquatic Biology and Toxicology, Department of Biosciences, University of Oslo, 0371 Oslo, Norway; Center for Biogeochemistry in the Anthropocene, University of Oslo, 0371 Oslo, Norway; Faculty of Environmental Sciences and Natural Resource Management, Norwegian University of Life Sciences, NMBU, 1432 Ås, Norway; Section for Aquatic Biology and Toxicology, Department of Biosciences, University of Oslo, 0371 Oslo, Norway; Center for Biogeochemistry in the Anthropocene, University of Oslo, 0371 Oslo, Norway

**Keywords:** microbial metabolism, thermodynamics, emission, soil, lake, arctic

## Abstract

Redox conditions, influenced by the availability of oxygen, are expected to dictate the rate of CO_2_ and CH_4_ production and to shape the composition and metabolism of microbial communities. Here, we use thawing permafrost peat in thermokarst water under a gradient of initial O_2_ concentrations to experimentally cover the variability in redox conditions potentially found across thawing landscapes. The three main greenhouse gases, CO_2_, CH_4_ and N_2_O, responded differently to O_2_ absence. CO_2_ production along the O_2_ gradient could be modeled by the Michaelis Menten equation revealing a sharp decrease when oxygen dropped under 100 μM. Under anoxic conditions CO_2_ yield decreased by 98% and maximum net production rate by 85% when compared to oxic conditions during the 11 days after thaw. N_2_O production was observed under anoxic conditions, while CH_4_ yield and CH_4_ accumulation rates did not differ across the redox gradient. The latter is due to the release of stored CH_4_ due to thawing. Differences between oxic and anoxic conditions were reflected in the microbial genomic composition, with changes in taxonomic and functional groups, such as N_2_O reducers, fermenters, denitrifiers and sulfur reducers increasing under anoxic conditions. Genomic changes towards less efficient central metabolism further explained the CO_2_ production yields and rates limited by O_2_ availability as predicted by thermodynamics. Together with the Michaelis Menten models the metabolic reconstruction pinpoint to critical thresholds of CO_2_ release at suboxic conditions and thus need to be considered when explaining and modeling highly variable CO_2_ emissions across thawing landscapes.

## Introduction

Redox reactions are central to the basic functions of life, including catabolic to anabolic reactions [1]. These metabolic reactions are also an important regulator of aqueous biogeochemistry because the oxidation state of elements affects their solubility (e.g. [2]), adsorption behavior (e.g. [3, 4]), toxicity (e.g. [5]), and distribution (e.g. [6]). By affecting redox conditions (RCs) redox reactions modify the environment, leading to a redox cascade that follows a repeatable succession based on the free energy of the reactions (ΔrG) [7]. As metabolic pathways change, this leads to different mineralization end products, e.g. in the form of greenhouse gases (GHGs).

It is well known that microbial communities organize according to the redox tower—the ranking of half-reactions by electrochemical potential [7–9]. This is based on Odum and Pinkerton [10], who extended the ideas of Lotka [11] within a thermodynamic framework and proposed that the efficiency of metabolism in organisms is optimized to maximize energy conservation. While the organization of redox reactions can be predicted, little is known about how rates of biodegradation, composition of metabolic pathways, and rates of GHG production are connected under different RCs in complex natural microbial communities. This is stunning considering that changes in RC have been observed in response to anthropogenic disturbances such as eutrophication [12], salinification [13, 14], changed hydrological conditions [15], cryosphere thaw [16], and other human-induced impacts in aquatic environments.

Thawing permafrost landscapes are candidates for important positive feedbacks to anthropogenic warming and can complicate the path to reach climate mitigation targets [17, 18]. The outgassing of GHGs from permafrost containing landscapes are of global significance [19, 20] and these emissions are expected to double this century [21, 22]. In the northern hemisphere, between 15% and 22% of the terrestrial surface is underlain by permafrost, of which 8%–12% are permafrost-affected peatlands [23, 24]. Permafrost peatlands cover ~1.7 million km [23, 24] and store about one-third (~185 Pg C) of the carbon (C) locked up in permafrost [25]. These estimates are highly uncertain since there is still limited knowledge on how this sequestrated C will be released to the atmosphere, with environmental conditions such as temperature, water content, nutrients and vegetation implicated in determining the fate.

Many peatlands have acted as a long-term C sink in the Holocene [26], but warming alters the C cycle in these landscapes and likely turns these ecosystems into sources of C, mainly in the form of CO_2_ and CH_4_, but also N_2_O [27] to the atmosphere. Observations in northern Norway point to degrading permafrost peat plateaus as sites of abundant methanogens and high rates of methanogenesis, particularly during the thermokarst pond stage [28]. Yet, quantifying GHG emissions from northern permafrost areas remains challenging due to the variability of these thawing landscapes ranging from waterbodies to drier and wetter soil conditions [29, 30] that determine the amount and form of GHG release to the atmosphere.

The degradation of permafrost may happen in uplifted dome-shaped landscape features (palsa/peat plateaus) that are drained from water leading to oxygenated (i.e. O_2_ saturated) conditions. Permafrost may also thaw in expanding thermokarst lakes and wetlands (palsa mires) where steep gradients in RC, including O_2_ depleted conditions, exist and large amounts of methane are released [31, 32]. Dynamics of the thawing permafrost may change the RC by temporarily causing more water-logged soils (or peat) with oxygen-depleted conditions, followed by drying through drainage leading to reoxygenation, exemplified by the widespread loss of tundra ponds [33]. Increased temperature and rainfall may also affect the water balance in high-latitude areas [34], which is expected to strongly affect the RC and oxygen availability, and thus microbial processes.

Understanding the microbial responses to the climate change-driven transformation from frozen peat to thermokarst and new peat is important to constrain C cycling including the potential transformation of the arctic landscapes from sinks to sources of C [35, 36]. Having a detailed metabolic understanding is a prerequisite for predicting future C balances. Including CO_2_, CH_4_ and N_2_O release rates and their respective warming potential in these ecosystems is also crucial for improved parameterization of Earth system models and thus modeling future climate. While numerous gradient studies exist on what constrains GHG emissions based on statistical relationships [37] experimental validation of the main drivers, such as RCs including oxygen availability, is limited [38] and a detailed understanding of the metabolic underpinnings is mostly lacking.

Here, we inundated a permafrost peat obtained from a collapsing peat plateau in water from an adjacent thermokarst pond that contained microbes with both aerobic and anaerobic metabolism. We used this blend as an experimental model system to study GHG production under different RCs (from O_2_ saturated to depleted conditions) potentially imposed by spatial and temporal dynamics in thawing landscapes [39]. The main objective of the present study was to quantify C mobilization (as changes in DOC) and mineralization potentials (as approximated by CO_2_ production) in water-saturated thawing permafrost peat under different RCs at 10 degrees and model CO_2_ production and yield using the Michaelis Menten equation (a first-order differential equation). For this, we determined post-thaw CO_2_ and N_2_O production in thawing permafrost peat in response to variable RCs (aerobic to anaerobic) *ex situ* at high temporal resolution over 11 days and linked these to the genomic properties of the microbial community. We also attempt to quantify the release of CH_4_ from thawing peat and CH_4_ oxidation potential under oxic and anoxic conditions after thawing. While the experiment is based on samples from a single site, it experimentally mimics corresponding oxygen gradients in soil, peat and lakes, and demonstrates generic properties of microbial metabolism and transformations along redox gradients forming in terrestrial to aquatic environments.

## Materials and methods

### Site description, environmental sample collection, and permafrost characteristics

Permafrost samples were collected from a peat plateau at Iškoras, Finnmark, Northern Norway (69°20′27” N, 25°17′44″ E) in September 2020. Lakewater samples were collected from a thermokarst pond close to the permafrost site, (69°20′27” N, 25°17′44″ E) to have a bacterial inoculation source and simulate permafrost degradation under thermokarst conditions. Sample collection has been described previously [40]. In short, a permafrost core was collected when the active layer above the permafrost reached its maximum depth. After the active layer was removed and put on a tarp using a shovel a steel pipe with a 30 mm inner diameter was hammered into the permafrost layer vertically, sampling frozen peat in ~5 cm increments until the mineral soil layer was reached. Details on peat chemistry and water characteristics can be obtained from [40].

### Sample preparation and experimental setup

**Figure 1:**
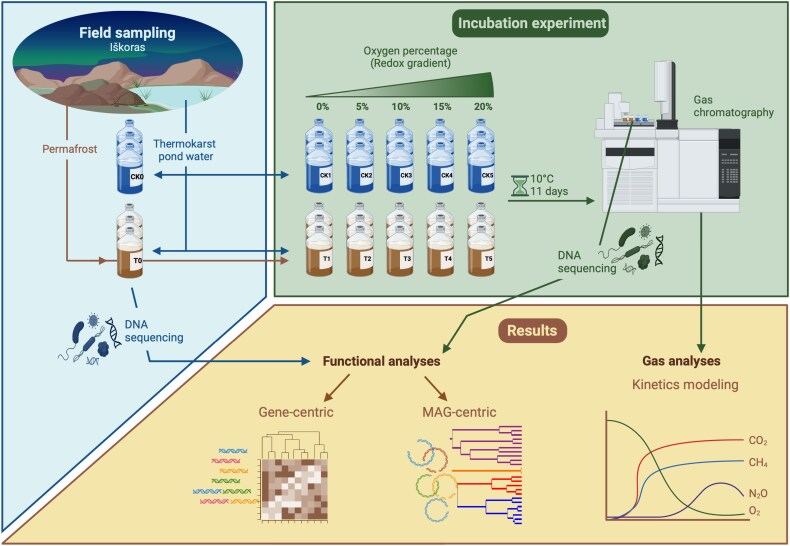
Outline of the experiment including sample acquisition of permafrost and thermokarst lake water samples for the batch culture incubations (**A**), the experimental design and robotics set up (**B**) and results from continuous gas measurements (CO_2_, N_2_O, CH_4_ and O_2_) and gene-centric and MAGs based metabolic reconstructions (**C**). Created in BioRender. Carlsen, E. (2025) https://BioRender.com/c22j381

Incubations were set up as a factorial experiment with different initial O_2_ headspace concentrations (4.5, 90, 170, 255 and 446 μM O2) and permafrost peat addition as the two factors (Fig. 1; Supplementary Table S1). Samples were incubated as stirred soil slurries diluted with thermokarst pond water. The 2.7 g (dry-weight) to 60 ml peat-to-lake water ratio was chosen to eliminate physical constraints for substrate availability and microbial growth and simulate water-saturated conditions. Incubations were divided into two groups, the treatment group with permafrost peat (from T1 to T5) and the control group (from CK1 to CK5) without permafrost peat to ensure that responses were peat addition dependent. Samples at the start of the experiment (T0 and CK0) were used to characterize the temporal development in the batch cultures.

Permafrost peat from 86–97 cm depth below surface was chosen for the incubation experiment according to the DOC-depth profile [40] to reach the highest possible DOC amount in the incubation system. The frozen peat sample was thawed in a helium atmosphere for 15 hours at 4°C before transferring ~2.7 g of the thawed peat to fifteen serum flasks (120 ml) each. Another 15 flasks were prepared without peat, serving as controls to enable assessment of the effects resulting from the permafrost addition. All flasks received 60 ml of lake water (see Supplementary Table S1) and a magnetic stir bar. This enabled homogenized mixing minimizing the establishment of major diffusion gradients, microenvironments and metabolic variability in the flasks. The serum flasks were crimp-sealed with butyl rubber stoppers and aluminum caps.

All flasks were He-washed by three rounds of evacuation and He-filling while stirring, using an automated manifold. After adjusting headspace pressure to 1 atmosphere increasing amounts of pure O_2_ (medical grade, AGA, Norway) were injected to create a gradient of initial O_2_ concentrations in triplicates according to Supplementary Table S1. The flasks were placed immediately into a temperature-controlled water bath adjusted to 10°C mimicking the average summer temperature of the thermokarst ponds in the sampling area. The water bath was equipped with submersible stirring boards for continuous stirring and is designed as an automated incubator [41, 42]. Automatic headspace sampling is made possible by the robotic arm of an autosampler (GC-Pal, CTC, Agilent, California, USA) which pierces the flasks in regular intervals and extracts ~1 ml of headspace gas with a peristaltic pump (Gilson 222XL, Gilson, Wisconsin, USA) which is connected to the automatic admission system of a gas chromatograph (Agilent 7890A, Agilent, California, USA). The GC has two columns and three detectors (TCD, FID, ECD) for simultaneously determining O_2_, CO_2_, N_2_, CH_4_, and N_2_O concentrations. To keep the headspace pressure at ~1 atmosphere, the pump is reverted between analyses, replenishing sampled gas with helium. Dry flasks with standard mixtures of known concentrations (AGA, Norway) were included in the measurement sequences for calibration and for evaluating the dilution resulting from He dilution. He-filled bottles were included to evaluate the leakage of gases into the measurement system. Headspace gas concentrations were monitored every 6 hours for up to 268 hours (11 days) to capture gas kinetics after thawing at high temporal resolution. The incubation time was chosen to capture the initial post-thaw processes and thus were terminated when CO_2_ concentrations in all flasks reached a first plateau.

### Chemical analyses

At the start and end of the incubations, 1 ml was transferred to 2 ml centrifuge tubes with pre-added 100 μl 20% glutaraldehyde solution for subsequent flow-cytometric cell count measurements. The rest of the volume was collected into 60 ml syringes and pressed through Sterivex filter units (0.22 μm, Merck Millipore, Germany) to capture the microorganisms for DNA extraction and downstream analyses. Filtrate was kept in 50 ml falcon tubes to measure pH and dissolved nutrient content.

pH was measured using HACH H170 (Hach, USA) and dissolved organic C was measured by infrared CO_2_ detection after catalytic high-temperature combustion (Shimadzu TOC-VWP analyzer, Shimadzu, Japan). Total dissolved nitrogen was measured by detecting nitrogen monoxide by chemiluminescence using a TNM-1 unit attached to the Shimadzu TOC-VWP analyzer (Shimadzu, Japan). Dissolved phosphorus was measured on an autoanalyzer as phosphate after wet oxidation with peroxodisulfate.

### Microbial cell counts

Density centrifugation was used to separate microbial cells from the soil material [43]. Histodenz solution (60% W/V, Sigma Aldrich, Germany) was carefully injected underneath the samples fixed by glutaraldehyde solution and centrifuged at a speed of 14 000 g for 90 min. All layers above Histodenz solution were collected and stored at 4°C. Cells were stained with the fluorescent nucleic acid stain SYBR green I (Molecular probes, Invitrogen, Massachusetts, USA) for at least 30 minutes (1x final concentration) and were then counted with an Attune® NxT Acoustic Focusing Cytometer (Thermo Fisher, Massachusetts, USA) equipped with an Invitrogen Attune NxT Autosampler equipped with a 488 nm laser using green fluorescence for triggered particle scoring [44]. Cell counts were extracted using Attune Nxt Software (Thermo Fisher, Massachusetts, USA) Supplementary Fig. S9.

### Metagenomic sequencing

The 0.22 μm Sterivex cartridges were immediately frozen and stored in −80 freezers until further analyses. Total DNA was extracted using a DNesay PowerWater Sterivex Kit following the manufacturer’s instructions (Qiagen, Germany). Libraries were prepared from 20 ng input DNA using the Nextera DNA Flex reagents (Illumina, California, USA) according to the manufacturer’s instructions, with eight cycles PCR and unique dual index adapters. Library concentrations were determined by quantitative PCR using KAPA reagents (Roche, Switzerland) and a single equimolar pool of all libraries prepared. Sequencing was performed on a NovaSeq 6000 instrument using a S4 flowcell for a 150-bp paired-end run (Illumina, California, USA). This resulted in 3.39 × 10^9^ raw reads (9.4 × 10^7^ average per sample with a range from 5.7–17.5 × 10^7^; for details see Supplementary Table S2).

### Bioinformatic analysis

The raw, Illumina NovaSeq whole-genome shotgun DNA sequencing data were processed as outlined in the supplementary material resulting in metabolic functions of the microbial communities as reconstructed and analyzed using both a gene- and genome-centric approach. The code is available at https://gitlab.com/alper1976/marmip/-/tree/main/eira/papers/Redox_GHG_permafrost_thaw

### Gas analyses

The measured gas concentrations for CO_2_, CH_4_, O_2_, N_2_, and N_2_O were corrected for dilution, leakage and gaseous dissolution [41]. The resulting whole-flask CO_2_ kinetics were analyzed using the “growthrates” package in R, by fitting linear models to the period of maximum exponential increases to obtain the CO_2_ maximum gross production rates from each experiment, and for O_2_ maximum consumption rates and gross consumption were estimated. A two-parameter Michaelis–Menten kinetics model was then fitted to the CO_2_ maximum net production rates (v) over the redox gradient (O_2_ concentrations; S) using R package drc and its function drm based on the equation:


$$ \mathrm{v}={\mathrm{Vm}}^{\ast}\mathrm{S}/\left(\mathrm{K}+\mathrm{S}\right) $$

where Vm is the asymptote and K is halfway between 0 and the asymptote (Michaelis–Menten constant).

The respiratory quotient (RQ) was estimated as the changes in total concentrations of O_2_ and CO_2_, and the ratio between them, using linear models as implemented in R base.

### Ecoinformatic analysis

Rarefaction curves were computed using “rarecurve” from the vegan package [45] with outputs suggesting that gene and pathway annotations reached saturation and thus sequencing depth was sufficient for the gene-centric approach. Reads were normalized using trimmed mean of M values (TMM) through the edgeR package [46], which estimates scaling factors based on a reference sample from the present study, making it compositional. Alpha diversity indices were estimated from rarefied gene and module tables including functional richness with ACE, a nonparametric method for estimating the number of functions (KOs or KEGG modules) using sample coverage and Pielou’s evenness. For the genome-centric approach, the reads mapped to co-assembled contigs binned and dereplicated into metagenome assembled genomes (MAGs) were counted to build a MAG-per-sample feature table. Since MAGs were placed onto the GTDB phylogeny, we used the Faith’s Phylogenetic Diversity [47] index to measure and compare MAG diversity between treatments.

### Statistical analysis

For ordinations, non-metric multidimensional scaling in vegan (metaMDS function) was used with Hellinger transformation and Bray Curtis as the distance measure on the compositional matrices (beta-diversity measures) including annotations to taxonomy, KOs, traits and KEGG modules. Initial O_2_ concentrations were then fitted onto the ordination to obtain correlation coefficient and significance levels with beta-diversity. Differentially abundant traits were assessed using DESeq2 [48], contrasting the anaerobic treatments with each of the different oxic O_2_ concentrations, in addition to a comparison between all treatment samples and the control samples. The gllvm function of the gllvm package [49] was used to fit generalized linear latent models of the negative binomial type to responses of individual taxa, KOs, traits, and KEGG modules to the explanatory variable O_2_ in the permafrost addition experiment, with relationship significance determined from Wald statistics. For the genome-centric analysis, Phylogenetic-Robust Principal Component Analysis was done using *gemelli* [50] and QIIME2 [51] to identify clades associated with the different O_2_ treatments and use them for log-ratio calculation and to model these log-ratios along the O_2_ concentration gradient in the treatment samples. The latter was done by fitting a logistic relationship (of the form y ~ a-log(x + b) + c) in python using functions ‘curve_fit’ and ‘r2_score’ from scipy.optimize.curve_fit v1.11.4 [52] and sklearn.metrics v1.5.2 [53], respectively. The Faith’s Phylogenetic Diversity of these samples was also compared to the oxygen concentration levels, i.e. for five treatments with three replicates each, by fitting an exponential decay relationship (of the form y ~ a.exp(−xb) + c) using the previously mentioned python functions [52, 53]. We also measured the log-fold changes of each MAG present in at least six out of the 15 treatment samples using differential abundance analysis with Songbird [54], for models of each O_2_ level against the 0% O_2_ samples as reference.

## Results

### Permafrost degradation is influenced by redox

**Figure 2:**
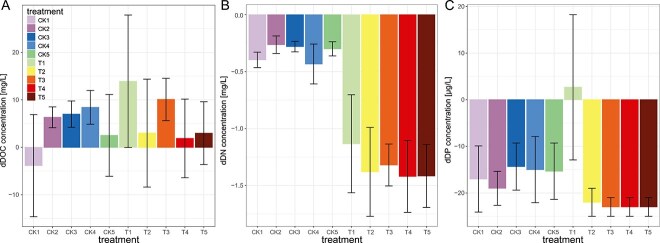
Changes in dissolved organic carbon (dDOC) (**A**), nitrogen (dDN) (**B**), and phosphorus concentrations (dDP) (**C**). Treatments with lake water are indicated by *CK* and lake water amended with permafrost by *T*; the numbers refer to the different O_2_ conditions (1: 4.5 μM starting O_2_ concentrations – 0.2%vol; 2: 90 μM O_2_–5%vol; 3: 170 μM O_2_–10%vol; 4: 255 μM O_2−_15%vol; 5: 446 μM O_2_–20%vol). Error bars show standard deviation and the number of replicates per treatment was three.

The experiment was designed to monitor post-thaw metabolic activity of permafrost peat under water saturated conditions. It is intended to experimentally test the effect of oxygen availability (redox) conditions on permafrost degradation, GHG production and the development of genome-encoded metabolism. At the start of the experiment DOC concentrations in the lake water incubations without permafrost (*CK* treatment) were 28.9 ± 1.1 mg C L^−1^. It should be mentioned that the starting conditions of the CK treatments closely reflect the conditions in the thermokarst pond water considering changes due to storage conditions. In the permafrost addition experiments (*T* treatment) initial DOC concentrations increased to 49.4 ± 0.9 mg C L^−1^. In the *T* treatments, DOC concentrations did increase in most treatments towards the end of the incubations resulting in DOC changes (dDOC) varying from −3.85 to 13.9 mg C L^−1^ (Fig. 2A). There were no significant differences in DOC and changes in dissolved N (dDN; Fig. 2B) observed in relation to the redox gradient. However, there was a significant decrease in DN across all treatments suggesting active N immobilization (i.e. mineralization and uptake) by microbes. Yet, changes in dissolved phosphorus (dDP) showed differences between oxic and anoxic treatments in the *T* (permafrost addition) treatments (Fig. 2C) with anoxic treatments showing no changes whereas in oxic treatments DP suggests active phosphorus uptake by microbes.

In comparison to the *T* treatments, the *CK* (thermokarst water) treatments showed an increase in DOC (positive dDOC) in all oxic treatments while under anoxic conditions dDOC was not different from zero. dDN (varying from −0.43 to −0.26 mg L^−1^) and dDP (varying from −19.67 to −14.33 mg L^−1^) values indicated microbial N and phosphorus immobilization in all water treatments. While dDP values in the *CK* treatments were similar to those of the oxic incubations with permafrost addition (varying from −24 to −22.33 mg L^−1^), dDN values were lower than in the permafrost addition (*T*) treatments (varying from −1.42 to −1.13 mg L^−1^); i.e permafrost addition induced larger changes in DN.

**Figure 3:**
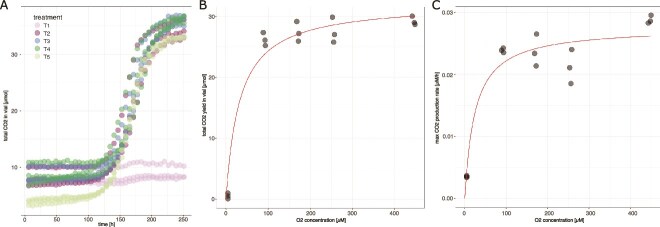
Changes in total CO_2_ and O_2_-dependent CO_2_ yield and production kinetics. (**A**) CO_2_ changes are plotted over time with color coding indicating the various treatments (1: 4.5 μM starting O_2_ concentrations – 0.2%vol; 2: 90 μM O_2_–5%vol; 3: 170 μM O_2_–10%vol; 4: 255 μM O_2−_15%vol; 5: 446 μM O_2_–20%vol). Michaelis Menten’s type of kinetics is given for CO_2_ yield (**B**) and maximum CO_2_ production rates (**C**). The number of replicates per treatment was three.

We used a Michaelis–Menten substrate-based kinetics framework to explore how CO_2_ production responds to O_2_ concentrations in freshly thawed permafrost peat incubated *ex situ*. Since pH change was small during the incubations (Supplementary Table S1), the CO_2_ release is likely due to mineralization. In the *T* treatments, the highly resolved changes in CO_2_ concentrations followed a sigmoid function in all incubations over time, and thus, showing expected lag phase, exponential phase and a leveling off (Fig. 3A). Estimating yields and maximum production rates from these curves revealed lower CO_2_ yield (or CO_2_ accumulation; Fig. 3B) and maximum production rates (Fig. 3C) when comparing oxic and anoxic conditions. CO_2_ yield under anoxic conditions amounted only 2.0% of the yield under oxic conditions. A fitted Michaelis Menten model suggests that changes in CO_2_ yield, which should correspond to the amount of organic matter being degraded throughout the incubations, occurred under suboxic conditions in treatments with less than 20 vol% initial O_2_ in the headspace or concentrations or initial dissolved O_2_ concentrations below 100 μM (Fig. 3B). Maximum yield was estimated to reach 32.2 μmol CO_2_ with an apparent half-saturation constant of 32.6 μM O_2_. Similarly, maximum production rates estimated from the sigmoid CO_2_ curves (Fig. 3A) showed an 85.4% decrease in maximum production rates under anaerobic conditions when compared to aerobic conditions (Fig. 3C). Maximum production rates were estimated to be 29.7 nM CO_2_ h^−1^ with half-saturation constant of 27 μM initial O_2_ using a Michaelis–Menten fitted model.

O_2_ consumption rates (Supplementary Fig. S1A) revealed the highest rates of 1.48 μM O_2_ h^−1^ in most O_2_-saturated treatments. Michaelis Menten’s type of kinetics fitted to the O_2_ uptake kinetics across the redox gradient suggested O_2_ limitations of aerobic respiration. Maximum consumption rates were estimated to be 4.03 μM O_2_ h^−1^ with a half-saturation constant of 790 μM O_2_ (Supplementary Fig. S1B). The respiratory quotient (RQ) as estimated from the changes in the concentrations of O_2_ and CO_2_ in the O_2_ amended flasks revealed values ranging from 0.43 to 0.58 with the lowest values in the most O_2_-saturated treatments (Supplementary Fig. S1C).

High-resolution CH_4_ data followed a logarithmic increase in the *T* treatments (Supplementary Fig. S2A). The high-resolution temporal dynamics did not differ across the O_2_ treatments with CH_4_ yield being indistinguishable. In the *CK* incubations, CH_4_ concentrations were below or close to the detection limit of the method. CO_2_ yield in the oxic *CK* treatments was between 7.4 to 10.6 μmol and between 3.5 to 4.1 in anoxic treatments. Maximum production (respiration) rates were similar among the *CK* treatments with a rate of around 1.6 nM CO_2_ h^−1^ (0.62–3.55), and thus an order of magnitude lower than those of the *T* treatments.

All three anoxic replicates showed net N_2_O production after 200 hours of anoxic incubations accumulating a maximum of 3 nmol N_2_O (Supplementary Fig. S2B). In one replicate, a decrease in N_2_O could be observed towards the end of the experiment indicative of microbial N_2_O reduction. In all other incubations including the *CK* treatments, N_2_O concentrations remained around background concentrations of the respective treatment.

Microbial cell numbers at the start of the incubation experiments were 2.5 ± 0.9 × 10^5^ cells ml^−1^ in the *CK* treatments and 1.04 ± 0.15 × 10^6^ cells ml^−1^ in the *T* treatments. While there were no significant differences along the redox gradient in either *CK* or *T* treatments, numbers increased until the end of the experiment by a factor of 2.3 to 5.7 ± 1.7 × 10^5^ cells ml^−1^ in *CK* treatments and by a factor of 3.6 to 3.78 ± 1.05 × 10^6^ cells ml^−1^ in the *T* treatments.

### Metabolic variations show differentiation along the experimental redox gradient

To assess taxonomic, compositional, and functional changes across the treatments, we applied a shotgun metagenomics approach from samples obtained at the incubations’ start (n = 6) and end (n = 30) points. We co-assembled (across treatment triplicates) 3.34 × 10^9^ high-quality reads from the 36 metagenomic samples (see Supplementary Table S2), resulting in 3.31 × 10^7^ contigs with a range per sample from 1.6 × 10^6^ to 3.6 × 10^6^ contigs and an N50 of 1364 (see Supplementary Table S3 and S4). These contigs captured ~74% (range 66 to 84%) of the reads per treatment, mapping 2.48 × 10^9^ reads (average 2.06 × 10^8^ per treatment with a range from 1.54 × 10^8^ to 2.74 × 10^8^ reads. Most domain annotated reads were of bacterial origin (on average 94 ± 3.6% of the reads), while 4.5 ± 3.65% were of eukaryotic, and 0.63 ± 0.53% of archaeal origin (see Supplementary Table S5). Of the 5.93 × 10^7^ open reading frames (ORFs) that could be annotated to KEGG KOs, 3.72 × 10^7^ ORFs could be assigned to KEGG pathways such as C fixation (Calvin cycle and other fixation pathways), photosynthesis, oxidative phosphorylation and CH_4_, N and S metabolism (for more details on data processing results see Supplementary Table S6).

In the gene-centric analysis, we focused on the permafrost amendments. These *T* treatments revealed a decrease in functional richness with increasing O_2_ availability while evenness did not vary significantly (Supplementary Fig. S3). Functional beta diversity, as determined by Bray Curtis distance, followed O_2_ concentrations according to PERMANOVAs performed on gene (R^2^ = 0.33, F-stats = 6.25, *P* < .005), and pathway (R^2^ = 0.37, F-stats = 7.80, *P* < .001) annotations (see Supplementary Table S7 for details on alpha and beta diversity statistics). Oxic treatments were not corresponding to O_2_ concentrations, and thus were indistinguishable in their functional profiles (Supplementary Fig. S4).

Comparison of the functional profiles between time zero (*CK0* representing the composition in the thermokarst water samples) and the end of the experiment revealed pronounced changes in the *T* treatments while changes in the *CK* treatments were in comparison minor (Supplementary Fig. S4, Supplementary Table S8). Shifts in the oxic *T* treatments encompassed a proportional increase in methanotrophy, aerobic respiration, fermentation and the Entner Doudoroff pathway (a mostly aerobic, low ATP-yield pathway), while a decrease was observed in anaerobic respiration, denitrification and genes involved in methanogenesis. Although the gene coding for the final step of methanogenesis (*mcr* gene) was not observed. Fewer traits changed significantly in the anoxic *T* treatments, with all observed changes being positive, as seen in traits such as methanotrophy, fermentation and the Entner Doudoroff pathway (Supplementary Table S8).

*CK* starting conditions (*CK0*), representing the thermokarst lake sample, encoded a wide range of metabolism including in addition to aerobic respiration signals for fermentation and anaerobic respiration (Supplementary Fig. S5). At the end point CK treatments were characterized by less changes compared to the *T* treatments, with a uniform increase in denitrification genes involved in N_2_O reduction independent of O_2_ treatment. Decreases in adenosine 5′-phosphosulfate (APS) reduction to sulfate via the APS reductase (APR) and aerobic respiration were observed in oxic treatments. When compared to *CK* treatments, *T* treatment samples revealed an increase in functional traits like methanotrophy, aerobic respiration (ubiquinone biosynthesis) and the Entner Doudoroff pathway. A decrease in the *T* treatment samples was observed in traits such as denitrification, anaerobic respirations (menaquinone biosynthesis) and genes involved in methanogenesis (for details see Supplementary Table S8).

**Figure 4:**
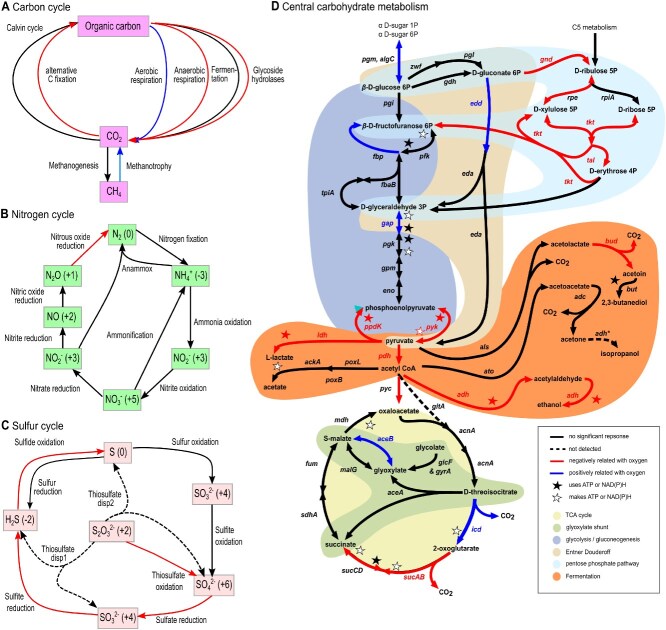
Functional patterns across the experimental redox (O_2_) gradient as revealed by metagenomics using a gene-centric approach. The fit of generalized linear latent models to responses of individual KEGG gene ontology terms involved in (**A**) carbon, (**B**) nitrogen, and (**C**) sulfur cycling as well as (**D**) central metabolism to the redox gradient. Significant relationships are determined from Wald statistics. Blue and red arrows represent significant positive and negative relationships with the experimental gradient. Detailed results from the generalized linear latent models are given in Supplementary Figs. S6 and S7, including estimated coefficients for predictors and their 95% confidence intervals.

After 11 days of incubations, we observed major differences between oxic (O_2_ concentrations >5 vol% or > 130 μM O_2_) and anoxic *T* treatments in individual key genes and pathways for C, N, and S cycling and energy metabolism. Using generalized linear latent models (gllvms), we found differential trait content along the redox gradient with e.g. alternative C fixation, anaerobic respiration, N_2_O reduction and glycoside hydrolases being negatively related to O_2_ (Fig. 4A-C). Furthermore, gllvms revealed an increase of methanotrophy, the glyoxylate cycle and Entner-Doudoroff pathway with O_2_ availability while the pentose phosphate pathway (both oxidative and nonoxidative phase), sulfate/sulfite/sulfide reduction, and N_2_O reduction were higher under anoxic conditions (Fig. 4A-D; Supplementary Fig. S6). These patterns were confirmed by detailed analysis of the KOs representing key steps of glycolysis, Entner-Doudoroff, and pentose phosphate pathways (Fig. 4D; Supplementary Fig. S7). Several key genes of ethanol, acetate, 2,3-butanediol, and lactate fermentation pathways were present in the incubations and mostly negatively related (*P* > .05) to O_2_ (Fig. 4D).

### Experimental redox gradient control on MAGs encoding various metabolic pathways

**Figure 5:**
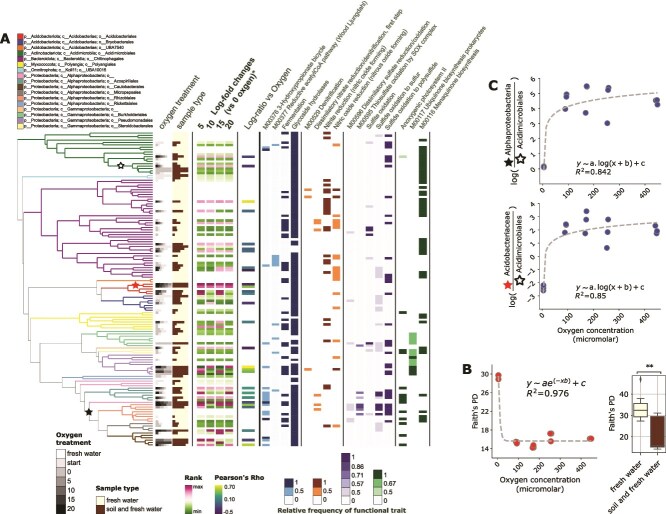
Metabolic potential and dynamics of MAGs in relation to the experimental redox (O_2_) gradient and phylogeny. (**A**) The phylogeny consists of the bacterial GTDB reference tree within which the MAGs were placed and classified using GTDB-Tk (branches are colored per taxonomic order). (**B**) The stars represent the specific MAGs used in the comparisons in panel (**C**) Barplots are used to show the (i) MAGs prevalence in different oxygen treatments (gray scale) and sample types (crème: Fresh water; brown: Soil and fresh water); (ii) log-fold changes in the >0% O_2_  *T* treatments measured using differential abundance in songbird; (iii) Pearson’s correlations of MAGs abundance (co-assembly vs one prevalent MAG, see Supplementary Table S4) and O_2_ concentration in the *T* treatments (min and max Pearson’s rho values indicated); and (iv) the relative frequency of sets of KEGG KOs that in each MAG are key to metabolic pathways (colored bars). A pathway is called in a MAG for frequencies ≥0.5 (see Methods). (**A**) Built with EMPress [82] in QIIME2 [51]. (**B**) Faith’s phylogenetic diversity along the O_2_ concentration gradient (left panel) with the dotted line representing the fit to an exponential decay relationship (for model fit see R^2^) and in the different sample types (right panel; Kruskal-Wallis test bracket; **: *P* < .001, *P* < = 0.01). (**C**) Log-ratios of selected taxa along the O_2_ concentration gradient in the treatment samples with dotted lines representing the fit to a logistic relationship (for model fit see R^2^). The fit was obtained for each of two log-ratios that considered Alphaproteobacteria (n108) and Acidobacteriaceae (n132) as numerators in ratios to Acidimicrobiales (n9). The names in parentheses represent the names of the clades on the Phylo-RPCA MAG tree (in panel **A**).

Subsequent binning of contigs resulted in 133 bacterial MAGs with 85% completeness and contamination below 5% (Fig. 5; Supplementary Tables S9). Similar to the gene-centric approach MAG read recruitment showed patterns corresponding with oxic and anoxic treatments, and associations specific to the permafrost addition (Fig. 5A). MAGs in the anoxic treatments with added permafrost (*T* treatments) were more similar to *CK* treatments and the starting conditions when compared to oxic incubations (Supplementary Fig. S8). This low functional turnover and the increase in phylogenetic diversity (Faith’s PD) when O_2_ was unavailable (Fig. 5B; fitted exponential decay) reflects the low activity in the incubations as reflected also by the low CO_2_ production rates and yields under anoxic conditions. There was also a high number of shared MAGs between the *CK* and *T* treatments. Some of these proliferated under anoxic conditions in the *T* treatments and encoded anaerobic respiration and fermentation potential (Fig. 5A), indicating that the thermokarst water sample included anaerobic bacteria. A few MAGs restricted to the anoxic *T* treatment, and thus can be regarded as permafrost peat specific.

MAGs specific, belonged to the Alphaproteobacteria and Acidobacteriae, and encoded capabilities of sulfate reduction and other S cycling genes. MAGs from these clades had high loadings along the first axis of the Phylo-RPCA separating the oxic and anoxic samples, and produced log-ratios values (using two prevalent Acidimicrobiales MAGs also enriched in the treatments as references) revealing an exponential increase in abundance at suboxic conditions (Fig. 5C). Interestingly, the Alphaproteobacteria MAGs possessed various N cycling (i.e. steps of denitrification) and exclusive sulfide oxidation capabilities, but not necessary those MAGs exhibiting highest log-fold changes in high O_2_ samples (Fig. 5A). MAGs associated with anoxic conditions (light grey in high-diversity O_2_ treatment samples) belonged to a wide range of phyla, with fermentation, denitrification (single MAG belonging to the Pseudomonadales and found in the anaerobic and lowest O_2_ treatment), C fixation (3-Hydroxypropionate bicycle and Wood-Ljungdahl pathway) and S cycling pathways encoded in their genomes (Fig. 5A). Fermentative metabolisms included acetate, ethanol, and 2,3-butanediol fermentation. There were also several MAGs that encoded for menaquinones and dissimilatory sulfate reduction/oxidation often found in the anoxic treatments.

Bacteria typically observed in freshwater lakes and ponds [55], such as MAGs from the genus *Polynucleobacter* (MAG33) and *Novosphingobium* (MAG120), encode traits such as aerobic respiration. While *Polynucleobacter* MAG33 was exclusively found in the *CK* treatments, *Novosphingobium* MAG120 was only present in the *T* treatments. A number of Planctomycetes MAGs (MAG51, 52, 54, 58, 84) encoded most steps of denitrification and nitrification. Although these MAGs resemble previously suspected methanotrophic Planctomycetes [56] and included genes encoding for formate oxidation, most key genes including pmoABC and mmoX for the conversion of CH_4_ to methanol were missing. These were the closest candidates of methanotrophic MAGs and no other MAGs containing the genes encoding for the conversion of CH_4_ to methanol and further to formaldehyde could be identified.

## Discussion

As expected, RCs determine metabolic reactions with microbial communities organized according to the redox tower—the ranking of half-reactions by electrochemical potential [7–9]. Our experimental results show that the thermodynamics of the reduction/oxidation reactions dictate DOC mineralization and net production of GHGs. This confirms that environmental constrains of substrate supply (such as O_2_) have a vast potential to mask temperature dependence of enzymatic processes leading to GHGs [38, 39]. Oxygen gradients, and resulting RCs, can be observed in highly spatiotemporal variable thawing landscapes [57, 58], and thus need to be considered.

We present Michaelis–Menten-based models that allow us to predict CO_2_ production along different O_2_ concentrations. These Michaelis–Menten-based models suggest non-linear relationships between oxygen availability and GHG productions, and predict that at suboxic conditions, observed widely throughout thawing landscapes [57, 58], abrupt transition in metabolism occurs determining CO_2_ production yields and rates. These abrupt changes in CO_2_ production, and the associated Michaelis–Menten half-saturation constant, were accompanied by the development of taxonomic and functional diversity specific to O_2_ availability, particularly changes in the relative abundances of microbial C, N and S cycle genes. Reconstructions of MAGs further provide the metabolic underpinnings of this threshold according to the redox tower [7–9] and provide explanations for the efficiency of metabolism to maximize energy conservation [10] under variable RCs.

### Explanations for the lower CO_2_ production and DOC mineralization under anoxic conditions

The much lower CO_2_ production in anoxic incubations are likely due to anaerobic respiration and fermentation yielding less energy and producing less CO_2_ per organic C since organic fermentation products and/or H_2_ likely accumulated. It may also be due to the more recalcitrant nature of the organic matter under anoxic when compared to oxic conditions as indicated by low organic matter dissolution in anoxic treatments. For example, the degradation of both lignin and (hemi)cellulose under oxic conditions relies on oxidative enzymes, while under anoxic conditions microorganisms utilize sophisticated enzyme systems for coordinated but less efficient breakdown [59].

Interestingly, microbial cell numbers did not differ among the treatments which would have been expected from the CO_2_ production rates. Potential explanations are the masking by either inactive or dead cells introduced by the permafrost addition [60], as indicated by the marked (factor 4) increase in cell numbers due to permafrost addition at starting conditions, or differences in cell size (or cell specific biomass) across the treatments. In addition, less grazing by protists or predatory bacteria [61] could result in lower community turnover under anoxic conditions and thus result in similar standing stock of the microbial biomass under various oxygen concentrations.

The insignificant DOC changes in the anoxic *CK* treatments, indicative for very low organic matter dissolution, further supports the almost 50 times decrease in CO_2_ yield after 11 days in the anoxic when compared to oxic incubations. This is above the upper range of previous observations suggesting that anoxic environments typically emit 3.4 times less total C than oxic environments [58, 62]. Such ratios are likely influenced by the length of incubation experiments, physicochemical properties, and substrate characteristics [63]. A high contribution of chemolithoautotrophic processes to microbial biomass production can be another explanation and finds support in our metabolic reconstructions and low RQ values.

RQ values around 0.5 (meaning low CO_2_ production in comparison to O_2_ consumption) as estimated in our incubations of thawing permafrost are extremely low, with previous estimates ranging from 0.5 to 2 in freshwater systems [64]. Likely explanations are that various members of the community metabolize reduced compounds such as organic matter with high H and low O content [64, 65], or perform chemolitho(auto)trophic metabolism. Nitrification, and sulfur and iron oxidation, consume O_2_ and produce less CO_2_, likely due to CO_2_ fixation with metabolic reconstructions corroborating the presence of alternative C fixation pathways. This striking departure from the commonly assumed and applied RQ of 1 supports previous studies demonstrating the strong impact of substrate and metabolic pathways on RQ [64, 65], and calls for caution when converting from consumed O_2_ to produced CO_2_ or vice versa. This should be particularly true in environments with strong redox gradients and regular switches in RCs such as a thawing cryosphere.

Michaelis–Menten-based kinetics modeling of O_2_ consumption rates across O_2_ concentrations suggests a limitation of aerobic respiration by O_2_ at even saturated concentrations as maximum consumption rates were not reached. This is contrary to the assumption that reactive O_2_ species increasing with O_2_ concentration may limit O_2_ consumption rates at high O_2_ concentrations. Moreover, the kinetics model supports previous observations that O_2_ can be exploited by high-affinity oxidases at very low concentrations, down to nanooxic conditions [66]. Under O_2_-depleted conditions, a shift to anaerobic respiration is corroborated by ubiquinones predominating under oxic and menaquinones under anoxic conditions [67], and an increase in genes encoding for sulfate/sulfite/sulfide reduction. In addition to changes in the electron transport chain and oxidation substrates, there are also adjustments to the central metabolism with central genes in the Entner Doudoroff [68] and Embden-Meyerhof-Parnas pathways being overrepresented under oxic while fermentative pathways and the pentose phosphate pathway were overrepresented at anoxic conditions. Besides the distinct yields of ATP and NAD(P)H, a lower number under anoxic than oxic conditions, these pathways theoretically result in a range of different metabolic intermediates and GHG yields for each molecule of sugar consumed, with the former awaiting to be confirmed by metabolomics.

### CH_4_ release besides missing CH_4_ cycling after the first days of thawing

In a GHG and climate change perspective, the production and fate of CH_4_ is most relevant, given that CH_4_ has 27.9 times more warming potential than CO_2_ on a 100-year timescale [69]. The a priori expectation of enhanced CH_4_-production by thawed permafrost peat under water saturated anoxic conditions was not confirmed in our study. This is most likely the result of that under short incubation (less than 2 weeks) periods, RCs might not lead to reduced conditions sufficient for methanogenesis to be thermodynamically favorable. Also, any potential biogenic production of CH_4_ is likely overwritten by the CH_4_ stored in the permafrost being released post-thaw (less than 1 nmol g^−1^ thawed dry-weight permafrost), as observed previously [40, 70].

That CH_4_ release was equal under oxic and anoxic conditions and thus across the entire experimental redox gradient after thawing, is supported by genomic data. Low numbers and no difference in gene abundances (*P* > .05) encoding for methanogenesis could be found across the RG. An additional explanation for methanogenesis not being detectable could be the inhibition of methanogenesis due to the pretreatment of the experiments leading to O_2_ exposure toxic for methanogens. Most likely syntrophic consortia between fermenters and methanogens need time (more than 11 days) to be established and thermodynamically favorable. The longer time for anaerobic consortia to be established provides a further explanation for the very low dissolution and CO_2_ productions yields and rates under anoxic conditions.

Besides the oversaturation of CH_4_ we observed a low abundance or lack of pmoA and mmoX abundance (pmmo and smmo) [71], as well as pXMO, encoded by the pxmABC operon, in most treatments which have also been observed in metagenomes from field samples. Our co-assembly-based reconstruction of MAGs resulted in representatives closely related to previously identified methanotrophs. However, annotations of these MAGs (including Planctomycetes, Verrucmicrobia, and Acidobacteria) did not reveal any genes involved in the conversion of CH_4_ to methanol and further to formaldehyde. This might be expected from methanotrophs being inactive in the frozen peat but rather unexpected as the thermokarst water included strong signals of methanotrophic potential. Still, we observed an increase in the methanotrophic potential by the end of the *T* treatments pointing to methanotrophs establishing themselves in the incubations although their activities were still not observable. This supports the notion of the low energetic yields of the reactions, resulting in very slow growth rates of the microbes responsible for CH_4_ oxidation [72].

Particularly in batch cultures as used in our experiments slow-growing microbes such as methanotrophs are likely not favored because of the long time required to establish a community capable of CH_4_ oxidation [73]. This might be different in natural settings where variable water levels can lead to variable redox states. Under variable conditions, community members adapted to the particular state might be always present and just need to be activated with the lag phase limited by transcription and translation, and thus a full establishment of a new community is not demanded. Still, microbes in permafrost thaw may act similarly to a batch culture where inoculation is provided by microbes from already thawed material.

### Minute amounts of N_2_O release under anoxic conditions by denitrifiers

Permafrost thaw has been suggested to cause localized N_2_O emission hotspots often in connection with rapid moisture changes [74, 75]. The role of soil moisture or water logging as a primary environmental control on N_2_O fluxes has been exemplified by the bell-shaped dependence of N_2_O fluxes on soil moisture peaking at the intermediate soil moisture range [27, 76]. Such intermediate moisture conditions likely provide the RCs for the two main microbial processes responsible for N_2_O production in soils: aerobic nitrification (oxidation of NH_4_^+^ via nitrite (NO_2_^−^) to NO_3_^−^, N_2_O as by-product) and anaerobic denitrification (reduction of NO_3_^−^ and NO_2_^−^ to gaseous N forms NO, N_2_O, and N_2_) [77].

Our results point to denitrifiers as potential N_2_O sources since the various steps of denitrification were overrepresented in the anoxic treatment. This is suggested by differential abundance analyses of the metagenomic data where denitrifiers were overrepresented (though not significantly at *P* < .05 in all cases but with negative coefficients across the various steps; see Fig. 4B and Supplementary Fig. S5) in the anoxic when compared to the oxic treatments. The decrease of N_2_O in one anoxic replicate towards the end of the incubation points to ongoing N_2_O reduction. This is also corroborated by the overrepresentation of N_2_O reduction genes in the anoxic treatments. As such our experiment highlights low potential of N_2_O emissions and the importance of denitrification and N_2_O reducers for net N_2_O production under anoxic conditions in thawing permafrost peat.

### Metabolic tuning in response to redox can lead to large variability in GHG emissions across thawing landscapes

Our genomic analysis showcases a distinct metabolic tuning to RCs, determining the transition from aerobic respiration to anaerobic respiration and fermentative metabolism, and associated highly different GHG yields and production rates. Besides the low production estimates leading to longer mineralization times under anoxic conditions, the increased functional richness, as shown by our results, under thermodynamically less favorable, anoxic conditions highlights the adaptability of permafrost communities to different electron acceptors and the potential of GHG production. Considering the highly dynamic nature of thawing landscapes, in particular the creation of new waterbodies and their drainage due to collapsing palsas and permafrost, it is not surprising to see highly variable GHG emissions across space and time. These small waterbodies residing on permafrost over large geographical scales in Eurasia and North-America are currently among the most vulnerable water-bodies globally [34, 78]. By their sheer number these systems may also serve as increasingly important conduits of GHGs and historical soil C stocks to the atmosphere [31, 32].

Although waterbody formation and expansion increase C fluxes to the atmosphere it is the RCs that determine how fast and at what warming potential these frozen soils get mineralized as shown in our study. The observed lag phases, in particular the long time that it takes for the establishment of CH_4_ cycling community members to emerge, emphasize the importance of future temporal studies to understand GHG dynamics under moisture changes and associated RC transitions.

### Shifts from aerobic to anaerobic metabolism drive tipping points in GHG emissions

From a theoretical perspective, the observable permafrost microbiota’s “aerobic” and “anaerobic” taxonomic states appear to be community-level phenotypes, traceable through specific taxonomic groups and community members (MAGs). These coalescing communities likely exhibit internal metabolic interdependencies challenging established notions of group-level individuality and “organismality” [79, 80]. This is exemplified by the presence of N_2_O producers (denitrifiers) and consumers (reducers) which are consecutively active as suggested by N_2_O concentrations in our anoxic treatments. The presence of fermentation potential but missing methanogenic activity further emphasizes the need to study anaerobic communities in more detail, since they experience thermodynamically less favorable conditions, and engage in syntrophic interactions [81].

An experiment has its drawbacks, as there are artificial aspects to it, including bottle and batch culture effects, and in our case metabolic limitation in the permafrost and the inoculum may lead to communities different to those in nature. While the mixing of thermokarst pond water and thawing permafrost may not represent a common natural phenomenon, the described outcomes of our redox gradient experiment should hold true across thawing landscapes. An advantage of this type of batch cultures is that they can reveal the establishment of coalescing communities and their dynamics under successional conditions, where they may appear to be “interacting as internally integrated units rather than as a collection of species that suddenly interact with another collection of species” [79]. Resulting group-level stability could promote non-linear responses and lag phases when changing RCs hit a threshold, as shown by our kinetic models. As such our experiment exemplifies by simulating cryosphere thaw [16] how anthropogenic disturbances [12–15] could result in highly variable GHG emissions even under minor redox changes.

## Supplementary Material

Supplementary_information_v6_ycaf009

## Data Availability

The raw demultiplexed metagenomic sequence data generated in this study have been deposited in the Sequence Read Archive under BioProject accession code PRJEB72786. Gas data, key database files, metagenomics and ecoinformatics configuration files and python/R notebooks are available at https://gitlab.com/alper1976/marmip/-/tree/main/eira/papers/Redox_GHG_permafrost_thaw.
