# Correction to: Redox determines greenhouse gas production kinetics and metabolic traits in water-saturated thawing permafrost peat

**DOI:** 10.1093/ismeco/ycaf111

**Published:** 2026-07-03

This is a correction to: Eira Catharine Lødrup Carlsen, Jing Wei, Franck Lejzerowicz, Sigrid Trier Kjær, Sebastian Westermann, Dag O Hessen, Peter Dörsch, Alexander Eiler, Redox determines greenhouse gas production kinetics and metabolic traits in water-saturated thawing permafrost peat, *ISME Communications*, Volume 5, Issue 1, January 2025, ycaf009, https://doi.org/10.1093/ismeco/ycaf009.

In the originally published version of this manuscript, the negative values in Figures 2B and 2C were depicted with an additional 1 instead of a `-', thus making the y-axis values wrong. Additionally, in two instances the unit was wrong in the text beneath Figure 2, showing dDP values in mg/l instead of the correct unit µg/L. These errors have been corrected.

