# Supplementary material for: Redox determines greenhouse gas production kinetics and metabolic traits in water-saturated thawing permafrost peat
Source: ISME Commun. 2025 Mar 3;5(1):ycaf009. doi: 10.1093/ismeco/ycaf009 (PMC11922181; doi:10.1093/ismeco/ycaf009)
Supplement: Supplementary_information_v6_ycaf009 [file supplementary_information_v6_ycaf009.docx]

**Supplementary information**

[Supplementary methods 2](#_Toc188361065)

[Bioinformatic analysis. 2](#_Toc188361066)

[Metabolic reconstructions 2](#_Toc188361067)

[Supplementary Table M1. Metabolic traits included in the analyses. 4](#_Toc188361068)

[Supplementary Table M2. Metabolic traits that were not included in the analyses. 7](#_Toc188361069)

[Supplementary tables 8](#_Toc188361070)

[Supplementary Table S1. Details on experimental set-up. 8](#_Toc188361071)

[Supplementary Table S2. Counts of raw reads per sample obtained by illumina sequencing. 9](#_Toc188361072)

[Supplementary Table S3. Counts of raw and mapped reads per treatment used in co-assemblies. 10](#_Toc188361073)

[Supplementary Table S4. Details on co-assembly. 10](#_Toc188361074)

[Supplementary Table S5. Statistics on taxonomic annotations. 11](#_Toc188361075)

[Supplementary Table S6. Statistics on annotations. 12](#_Toc188361076)

[Supplementary Table S7. Results from alpha and beta diversity analyses on KEGG KOs, Models and Pathways. 13](#_Toc188361077)

[Supplementary Table S8. Differentially abundant traits obtained through DESeq2 analysis. 14](#_Toc188361078)

[Supplementary Table S9. Details on Metagenome assembled genomes (MAGs). 17](#_Toc188361079)

[Supplementary figures 19](#_Toc188361080)

[Supplementary Figure S1. Changes in O_2_ concentrations, consumption kinetics, and respiratory quotient (RQ) along the experimental redox (O_2_) gradient. 19](#_Toc188361081)

[Supplementary Figure S2. Changes in the amount of CH_4_ and N_2_O over time in the various experimental redox treatments of the inundated permafrost. 19](#_Toc188361082)

[Supplementary Figure S3. Functional (KEGG KO) diversity across the experimental redox gradient. 20](#_Toc188361083)

[Supplementary Figure S4. Visualization of the functional diversity by non-metric multidimensional scaling (NMDS). 21](#_Toc188361084)

[Supplementary Figure S5. Heatmap of metagenome encoded traits in the thermokarst lake samples. 22](#_Toc188361085)

[Supplementary Figure S6. Results from a general linear latent model (gllvm) on traits. 23](#_Toc188361086)

[Supplementary Figure S7. Results from a general linear latent model (gllvm) on selected KEGG KOs involved in central metabolism. 24](#_Toc188361087)

[Supplementary Figure S8. Heatmap of metagenome assembled genomes (MAGs). 25](#_Toc188361088)

[Supplementary Figure S9. Example of gating strategy. 26](#_Toc188361089)

[References 26](#_Toc188361090)

# **Supplementary methods**

## **Bioinformatic analysis.**

The raw, Illumina NovaSeq whole-genome shotgun DNA sequencing data was first filtered to remove library adapter sequences, low-quality reads, and poly-G tails using fastp v0.23.244 (Chen et al., 2018), and the paired-end reads were merged using FLASh v1.2.1145 (Magoč & Salzberg, 2011). The filtered and merged reads were classified to the species of the GTDB reference database (version r207, release date: April 8, 2022) built to be used by Kraken2 v2.1.246 (Wood et al., 2019) and Bracken v2.747 (Lu et al., 2017). The resulting species classification was used to build a feature table expressed in read counts per sample and taxonomy tables for ecoinformatic analyses. The filtered and merged reads from treatment triplicates were co-assembled for using MEGAHIT v1.2.948 (D. Li et al., 2015). The quality of the assemblies was checked using metaQuast v5.2.049 (Mikheenko et al., 2016) and the as well as using the N50.sh script (https://github.com/hcdenbakker/N50.sh) to measure the number of scaffolds, assembly size, and N50/N90 values. The resulting contigs were directly annotated to KEGG gene ontology terms (KOs, also called K numbers), modules, and pathways using Prodigal v2.6.350 (Hyatt et al., 2010) and eggnog-mapper v2.1.951 (EggNOG DB v5.0.2) (Cantalapiedra et al., 2021). The contigs were binned for each co-assembly using MetaWRAP v1.452 (Uritskiy et al., 2018), which included a refinement step based on the contigs classified by three binners: CONCOCT v1.0.053 (Alneberg et al., 2013), MaxBin2 v2.2.654 (Wu et al., 2016) and MetaBAT2 v2.12.155 (Kang et al., 2019). The final refined bins (min 85 % completion; max 5 % contamination) were dereplicated into metagenome-assembled genomes (MAGs) across samples using dRep v3.4.056 (Olm et al., 2017) with a 95% average nucleotide identity measured using FastANI57 (Jain et al., 2018). The MAGs were classified and their quality was checked using GTDB-Tk v1.0.258 (Chaumeil et al., 2022), CheckM v1.2.259 (Parks et al., 2015), and CheckM2 v0.1.360 (Chklovski et al., 2023). For each sample, filtered reads were mapped to their contigs co-assemblies using Bowtie2 v2.3.561 (Langmead & Salzberg, 2012) and counted using SAMtools v.1.10 and Pysam v0.19.162 (H. Li et al., 2009). The gene and read counts associated with contigs or genes were grouped by function, by MAGs, or by function-per-MAG to obtain taxonomic and functional feature tables and later used in ecoinformatic analyses.

## **Metabolic reconstructions**

Metabolic functions of the microbial communities were reconstructed and analyzed using both a gene- and genome-centric approach. Gene-centric feature tables were built for the co-assembled contigs and the KOs and Module annotations of their predicted gene coordinates. To account for the multiple predicted traits per predicted gene, a normalization was conducted by dividing the number of reads mapping to this gene, and assigning this amount to each of the KEGG IDs, avoiding inflation of the total number of reads as proposed for mapping to multiple genomes (Zhu et al., 2021). The read counts for each individual trait were then grouped and summed, yielding features expressed in terms of coverage per sample. These features were then normalized using the method of trimmed mean of M-values (TMM), as recommended for gene abundance data analyses(Pereira et al., 2018). For the genome-centric features, a table with KO presence per MAG was constructed. We used the modules encoded in high-quality MAGs and coverage information to infer metabolic variations along the experimental redox gradient. Furthermore, KEGG annotations were used for functional analyses focused on C, nitrogen (N), and sulfur (S) cycling. The genetic potential of the microbial community was analyzed using KOs representing key C, N, and S marker genes for specific metabolic traits based on the methods used by (Lauro et al., 2009, 2011; Llorens-Marès et al., 2015; Panwar et al., 2020; Wei et al., 2023). These K numbers represent the KO (KEGG Orthologs) identifiers. The K numbers were collected from February to June 2024 from the KEGG PATHWAY, MODULE and BRITE database (Kanehisa, 2019; Kanehisa et al., 2023; Kanehisa & Goto, 2000). The following criteria had to be fulfilled for K numbers to be used in the assignment to a specific trait:

1. The K number must be unique for the specific pathway and/or module. K numbers occurring in multiple pathways are excluded.
2. Besides being unique to a pathway and/or module the K numbers must be represented by reactions being thermodynamically favorable in one direction (e.g. oxidation vs reduction). Therefore, reactions represented by arrows in the pathway maps pointing in only one direction are considered.
3. Where possible, the MODULE definition is the basis for the calculation of the metabolic trait. Where no MODULE definition is available, the information retained in the KEGG PATHWAY maps are the basis for the formulas used to calculate trait representation.

To avoid overestimation of traits, the following calculations were done:

1. Molecular complexes represented by more than one K number were divided by the number of K numbers representing said complex. This ensured that a molecular complex was only counted once, even though the complex was encoded by several K numbers (genes).
2. Traits consisting of subsequent steps were divided by the number of steps. This ensured that each trait was only counted once, also the traits consisting of several steps.
3. K numbers representing alternatives as indicated by comma separated K numbers in the MODULE definitions or in the KEGG PATHWAY maps were counted separately.

Any exceptions to these criteria are listed in the Supplementary table M1 (overview of the metabolic traits used in the analyses with relevant information) and M2 (overview of the metabolic traits that were not included due to lack of unique K numbers). All metabolic traits and MAG functional analyses were performed using python and R scripts (available at https://gitlab.com/alper1976/marmip/-/tree/main/eira/papers/Redox_GHG_permafrost_thaw)

## **Supplementary Table M1.** Metabolic traits included in the analyses.

*ID* represent the unique metabolic trait, while *Name*, *Entry* and *Definition* are collected from KEGG Module database and left empty if the trait was not listed in the Module database. The calculation of the metabolic trait is listed under *Formula*, and the *Enzyme (EC)*, *Genes*, *K numbers* and *Reaction* involved in each formula are collected from the KEGG database. Relevant information for each specific K number is listed under *Comment*, while information related to the metabolic traits is included under *Special remarks*.

## **Supplementary Table M2.** Metabolic traits that were not included in the analyses.

*ID* represent the unique metabolic trait, while *Name*, *Entry* and *Definition* are collected from KEGG Module database and left empty if the trait was not listed in the Module database. Relevant information for each specific K number is listed under *Comment*, while *Special remarks* highlight information related to the metabolic trait.

# **Supplementary tables**

## **Supplementary Table S1.** Details on experimental set-up.

Volumes and amounts of additions to individual flasks to set-up the treatments. This includes the creation of an oxygen gradient in the flasks by replacing headspace gas (He) with oxygen (O_2_). *T* indicates the lake water amended with permafrost while *CK* the unamended lake water treatment. The first number indicates the oxygen treatment with *1*: 0%; *2*: 5%; *3*: 10%; *4*: 15% and *5*: 20% O_2_ saturation, with *0* indicating the starting conditions. The second number indicates the replicate.

## **Supplementary Table S2.** Counts of raw reads per sample obtained by illumina sequencing.

*T* indicates the lake water amended with permafrost while *CK* the unamended lake water. *T* indicates the lake water amended with permafrost while *CK* the unamended lake water treatment. The first number indicates the oxygen treatment with *1*: 0%; *2*: 5%; *3*: 10%; *4*: 15% and *5*: 20% O_2_ saturation, with *0* indicating the starting conditions. The second number indicates the replicate.

## **Supplementary Table S3.** Counts of raw and mapped reads per treatment used in co-assemblies.

*“sample_name”* gives the samples, representing the triplicates of each treatment, used in each co-assembly. *“Reads”* indicates raw reads used in the co-assembly while *“mapped”* indicates the number of mapped reads to the total co-assembly. *“mapped_percent”* indicates the percentage of mapped reads. *T* indicates the lake water amended with permafrost while *CK* the unamended lake water treatment. The first number indicates the oxygen treatment with *1*: 0%; *2*: 5%; *3*: 10%; *4*: 15% and *5*: 20% O_2_ saturation, with *0* indicating the starting conditions. The second number indicates the replicate.

## **Supplementary Table S4.** Details on co-assembly.

*“sample_name”* gives the samples, representing the triplicates of each treatment, used in each co-assembly. Moreover, the number of contigs (scaffolds) are given as well as the assembly size, largest contig, N50, and N90 values per co-assembly. *T* indicates the lake water amended with permafrost while *CK* the unamended lake water treatment. The first number indicates the oxygen treatment with *1*: 0%; *2*: 5%; *3*: 10%; *4*: 15% and *5*: 20% O_2_ saturation, with *0* indicating the starting conditions. The second number indicates the replicate.

## **Supplementary Table S5.** Statistics on taxonomic annotations.

These numbers represent the mean (and standard deviation - *std*) of the taxonomic annotations on individual co-assemblies (triplicate samples from individual treatments). It includes domain annotations representing the counts and percentages of reads and contigs assigned to *Archaea, Bacteria, Eukarya*, and organelles as well as those that are unassigned (*unknown*). The average amount (and standard deviation - std) of contigs and reads assigned to the 3 domains and organelles.

## **Supplementary Table S6.** Statistics on annotations.

These numbers represent the statistics of the annotations on individual co-assemblies (triplicate samples from individual treatments) used for gene-centric analyses. It includes annotations to KEGG KOs and pathways representing the numbers and percentages of mapped reads and genes to assigned and unassigned genes, respectively. *T* indicates the lake water amended with permafrost while *CK* the unamended lake water treatment. The first number indicates the oxygen treatment with *1*: 0%; *2*: 5%; *3*: 10%; *4*: 15% and *5*: 20% O_2_ saturation, with *0* indicating the starting conditions. The second number indicates the replicate.

## **Supplementary Table S7.** Results from alpha and beta diversity analyses on KEGG KOs, Models and Pathways.

*Model* represent the formula of the analysis, the samples that were compared in Pairwise PERMANOVAs are showcased in *Pairwise contrasts*, while the statistical results are shown in *R2*, *F-stats*, *p-value* and *adjusted R2.*

## **Supplementary Table S8.** Differentially abundant traits obtained through DESeq2 analysis.

Only significant results are included in the table.

## **Supplementary Table S9.** Details on Metagenome assembled genomes (MAGs).

These numbers represent the statistics and taxonomic annotations of the high-quality MAGs (> 80% completeness and < 5% contamination; N = 133)

# **Supplementary figures**

## **Supplementary Figure S1.** Changes in O_2_ concentrations, consumption kinetics, and respiratory quotient (RQ) along the experimental redox (O_2_) gradient.

In panel A O_2_ changes are plotted over time with color coding indicating the various treatments (1: 4.5 µM starting O_2_ concentrations; 2: 90 µM O_2_; 3: 170 µM O_2_; 4: 255 µM O_2_; 5: 446 µM O_2_). Michaelis Menten's type of kinetics is given for O_2_ consumption (panel B) and C O_2_ concentration changes are plotted against O_2_ changes from which the respiratory quotient (slope of the relationship) was calculated (panel C).

## **Supplementary Figure S2.** Changes in the amount of CH_4_ and N_2_O over time in the various experimental redox treatments of the inundated permafrost.

CH_4_ (A) and N_2_O (B) changes are plotted over time with color coding indicating the various treatments (T1: 4.5 µM starting O_2_concentrations; T2: 90 µM O_2_; T3: 170 µM O_2_; T4: 255 µM O_2_; T-5: 446 µM O_2_).

**--**

## **Supplementary Figure S3.** Functional (KEGG KO) diversity across the experimental redox gradient.

Statistics of regression models for ACE richness (A) and Pielou´s evenness (B) are given. The shaded area represents the 95% confidence intervals of the models.

## **Supplementary Figure S4.** Visualization of the functional diversity by non-metric multidimensional scaling (NMDS).

NMDS plot based on Bray-Curtis dissimilarities derived from log-transformed KEGG profiles across the redox gradient. *CK* indicates lake water treatments and *T* permafrost addition treatments while the numbers indicate the O_2_ concentrations (1: 4.5 µM starting O_2_ concentrations; 2: 90 µM O_2_; 3: 170 µM O_2_; 4: 255 µM O_2_; 5: 446 µM O_2_). The last number indicates the replicate. T0 and CK0 represent the functional diversity at the start of the experiment. Stress value was 0.0246.


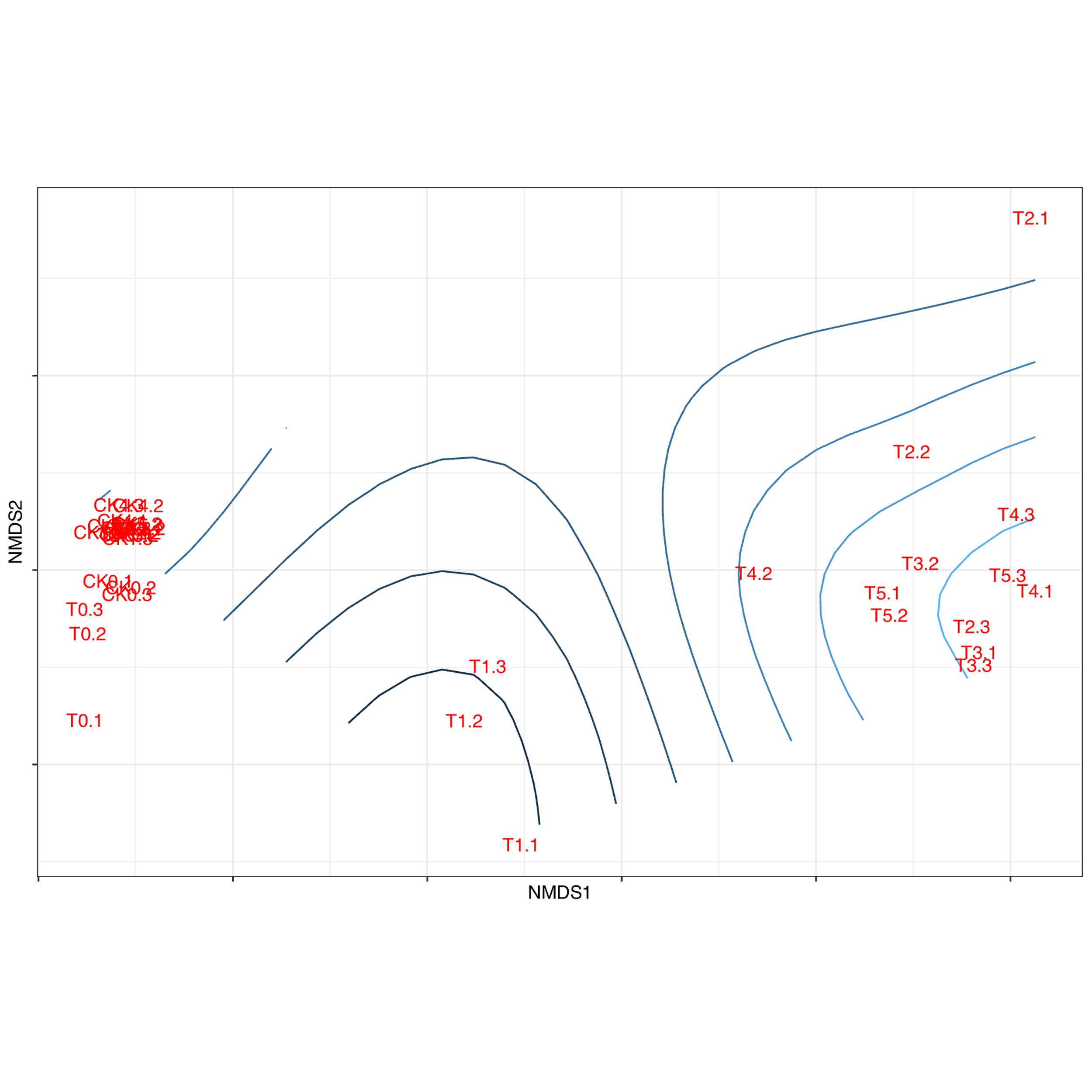


## **Supplementary Figure S5.** Heatmap of metagenome encoded traits in the thermokarst lake samples.

Heatmap showing the presence and absence of metagenome encoded traits in the thermokarst lake samples *(CK)* or so-called unamended lake water treatments at time zero.


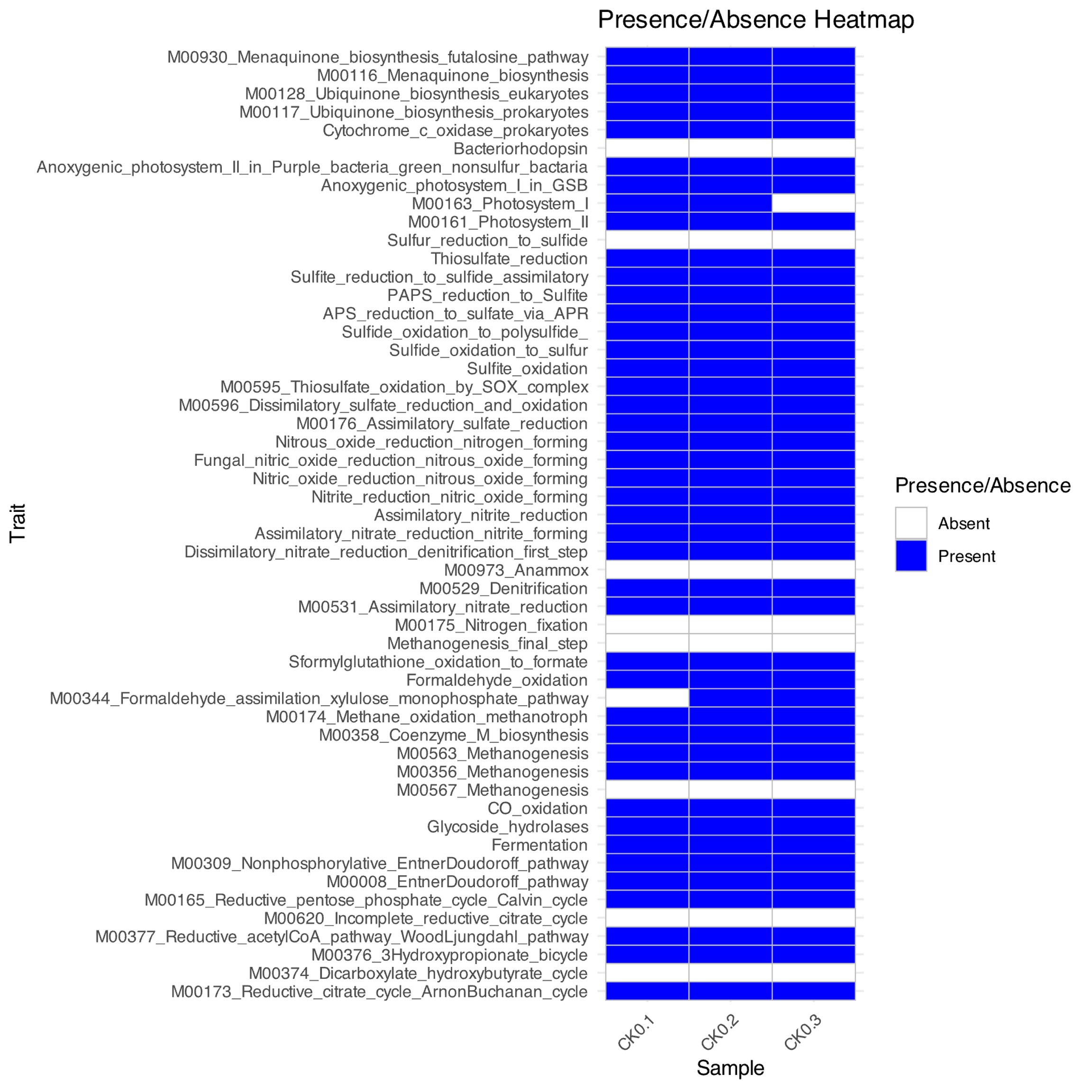


## **Supplementary Figure S6.** Results from a general linear latent model (gllvm) on traits.

Plots of Dunn-Smyth residuals, also known as randomized quantile residuals, are shown, including a plot of residuals against linear predictors of fitted values (A), a Normal Q-Q plot of residuals with a simulated point-wise 95% confidence interval envelope (B), residuals against row index (C) and column index (D) and scale-location plot (E). The estimated coefficients for predictors and their confidence intervals (F – redox gradient (O_2_ concentrations), allow us to study the nature of the effects of the experimental gradient on KEGG inferred traits. Point estimates (ticks) for coefficients of the environmental variables and their 95% confidence intervals (lines) are given, with those colored in black denoting intervals not containing zero.

-
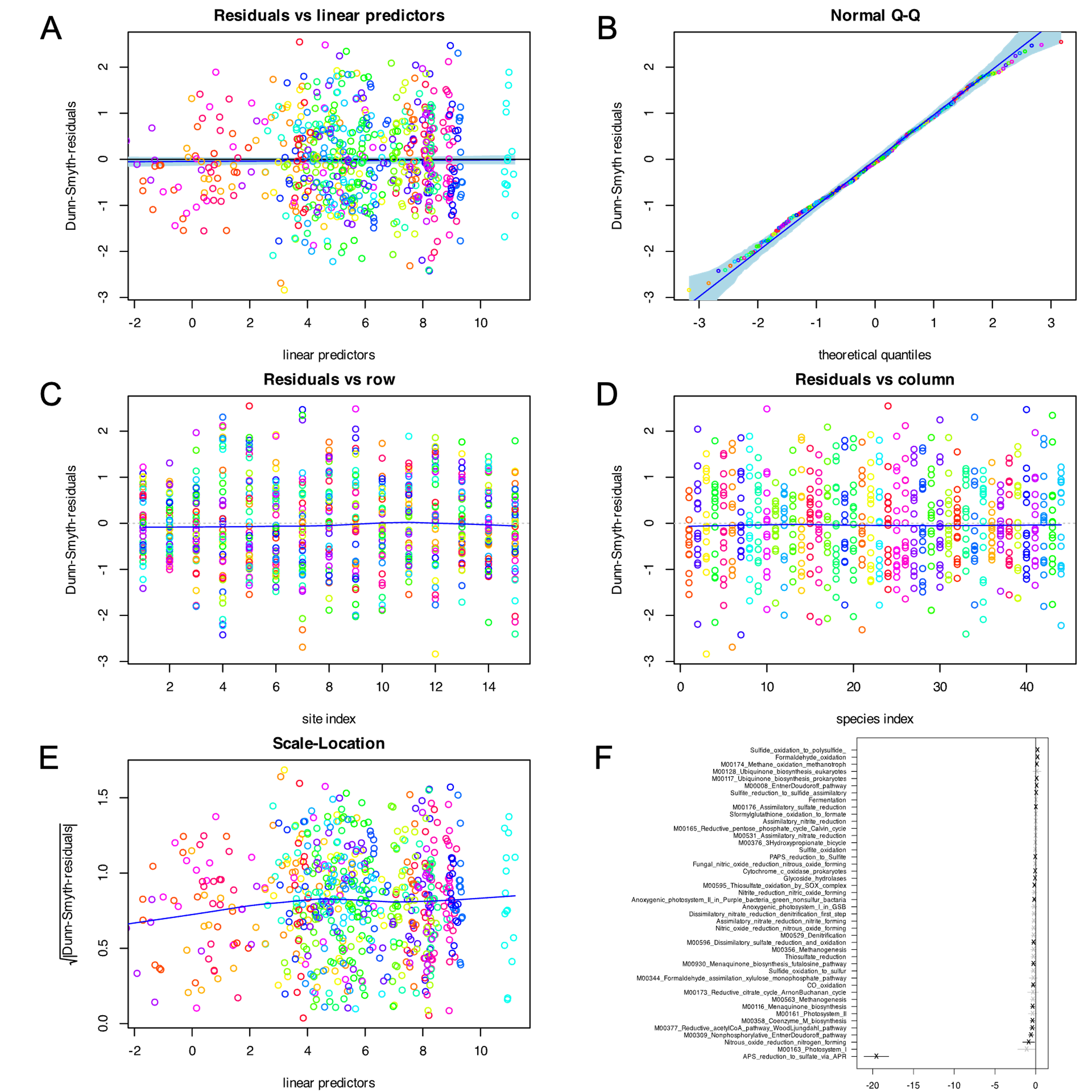


## **Supplementary Figure S7.** Results from a general linear latent model (gllvm) on selected KEGG KOs involved in central metabolism.

Plots of Dunn-Smyth residuals, also known as randomized quantile residuals, are shown, including a plot of residuals against linear predictors of fitted values (A), a Normal Q-Q plot of residuals with a simulated point-wise 95% confidence interval envelope (B), residuals against row index (C) and column index (D) and scale-location plot (E). The estimated coefficients for predictors and their confidence intervals (F – redox gradient (O_2_ concentrations), allow us to study the nature of the effects of the experimental gradient on KEGG KOs involved in the central metabolism. Point estimates (ticks) for coefficients of the environmental variables and their 95% confidence intervals (lines) are given, with those colored in black denoting intervals not containing zero.


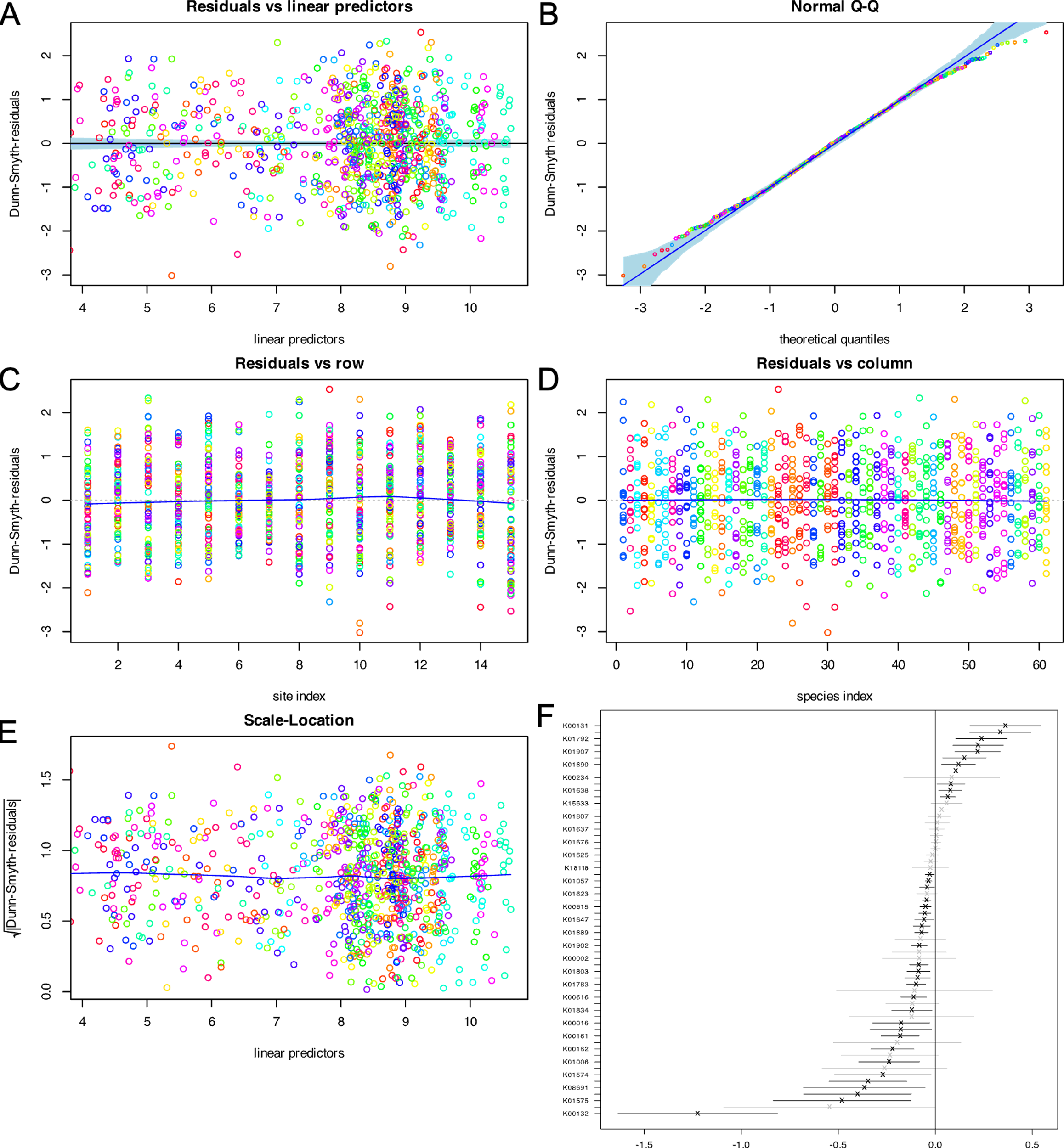


## **Supplementary Figure S8.** Heatmap of metagenome assembled genomes (MAGs).

Heatmap showing the presence of MAGs (columns) and the number of genes that they contain across the triplicated treatment’s co-assemblies (rows). *T* indicates the lake water amended with permafrost while *CK* the unamended lake water treatment. The first number indicates the oxygen treatment with *1*: 0%; *2*: 5%; *3*: 10%; *4*: 15% and *5*: 20% O_2_ saturation, with *0* indicating the starting conditions. The second number indicates the replicate.

## **Supplementary Figure S9.** Example of gating strategy.

The first populations of cells were distinguished based on their forward (FSC) and side scatter (SSC) properties. Next, single-parameter histograms and density plots were used on the fluorescence signals.


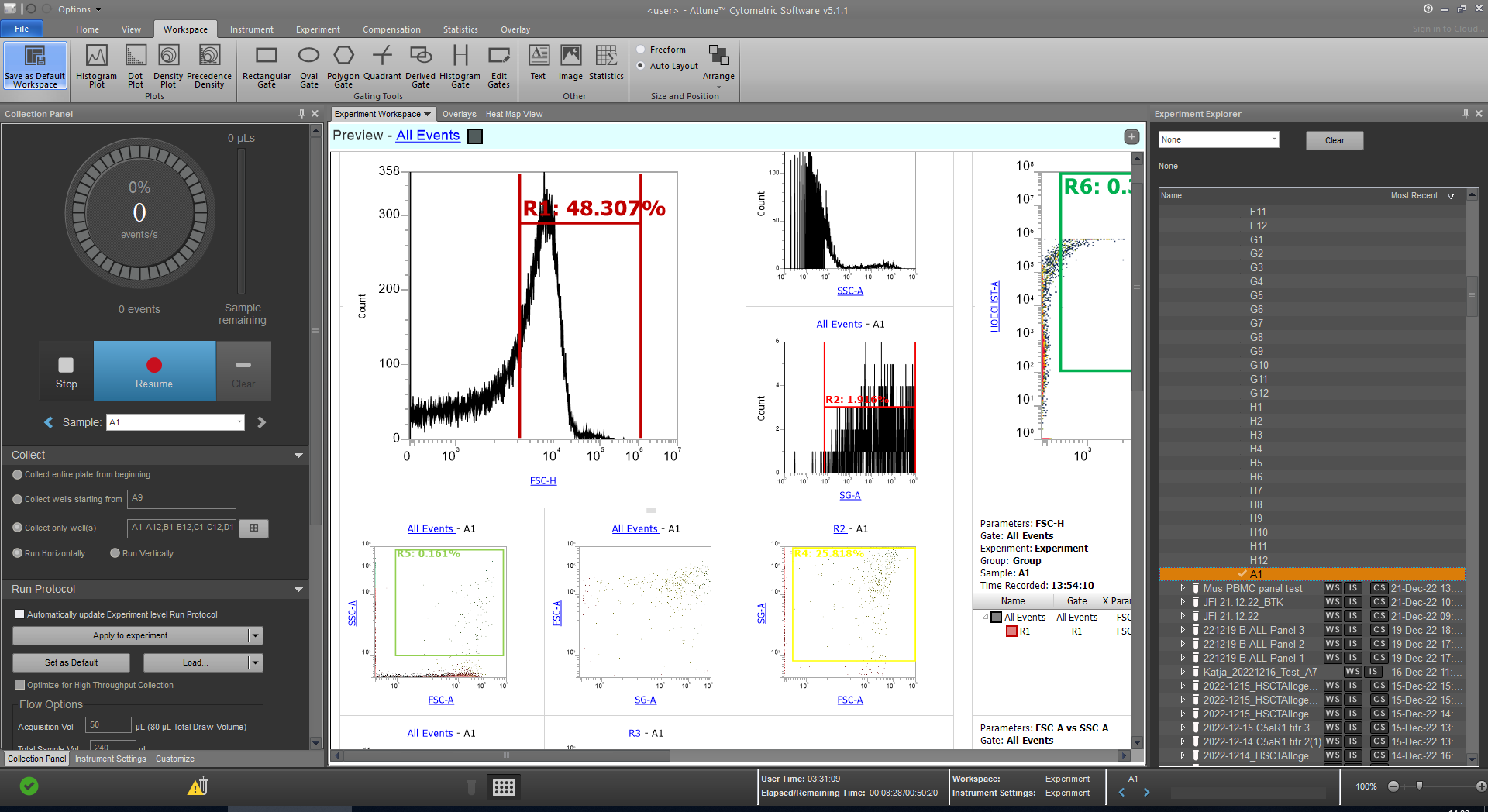


## **References**

Alneberg, J., Bjarnason, B. S., de Bruijn, I., Schirmer, M., Quick, J., Ijaz, U. Z., Loman, N. J., Andersson, A. F., & Quince, C. (2013). CONCOCT: clustering contigs on coverage and composition. *arXiv Preprint arXiv:1312.4038*.

Cantalapiedra, C. P., Hernández-Plaza, A., Letunic, I., Bork, P., & Huerta-Cepas, J. (2021). eggNOG-mapper v2: Functional Annotation, Orthology Assignments, and Domain Prediction at the Metagenomic Scale. *Molecular Biology and Evolution*, *38*(12), 5825–5829. https://doi.org/10.1093/molbev/msab293

Chaumeil, P.-A., Mussig, A. J., Hugenholtz, P., & Parks, D. H. (2022). GTDB-Tk v2: Memory friendly classification with the genome taxonomy database. *Bioinformatics*, *38*(23), 5315–5316.

Chen, S., Zhou, Y., Chen, Y., & Gu, J. (2018). fastp: An ultra-fast all-in-one FASTQ preprocessor. *Bioinformatics*, *34*(17), i884–i890. https://doi.org/10.1093/bioinformatics/bty560

Chklovski, A., Parks, D. H., Woodcroft, B. J., & Tyson, G. W. (2023). CheckM2: A rapid, scalable and accurate tool for assessing microbial genome quality using machine learning. *Nature Methods*, *20*(8), 1203–1212.

Hyatt, D., Chen, G.-L., LoCascio, P. F., Land, M. L., Larimer, F. W., & Hauser, L. J. (2010). Prodigal: Prokaryotic gene recognition and translation initiation site identification. *BMC Bioinformatics*, *11*(1), 119. https://doi.org/10.1186/1471-2105-11-119

Jain, C., Rodriguez-R, L. M., Phillippy, A. M., Konstantinidis, K. T., & Aluru, S. (2018). High throughput ANI analysis of 90K prokaryotic genomes reveals clear species boundaries. *Nature Communications*, *9*(1), Article 1. https://doi.org/10.1038/s41467-018-07641-9

Kanehisa, M. (2019). Toward understanding the origin and evolution of cellular organisms. *Protein Science*, *28*(11), 1947–1951. https://doi.org/10.1002/pro.3715

Kanehisa, M., Furumichi, M., Sato, Y., Kawashima, M., & Ishiguro-Watanabe, M. (2023). KEGG for taxonomy-based analysis of pathways and genomes. *Nucleic Acids Research*, *51*(D1), D587–D592. https://doi.org/10.1093/nar/gkac963

Kanehisa, M., & Goto, S. (2000). KEGG: Kyoto Encyclopedia of Genes and Genomes. *Nucleic Acids Research*, *28*(1), 27–30. https://doi.org/10.1093/nar/28.1.27

Kang, D. D., Li, F., Kirton, E., Thomas, A., Egan, R., An, H., & Wang, Z. (2019). MetaBAT 2: An adaptive binning algorithm for robust and efficient genome reconstruction from metagenome assemblies. *PeerJ*, *7*, e7359.

Langmead, B., & Salzberg, S. L. (2012). Fast gapped-read alignment with Bowtie 2. *Nature Methods*, *9*(4), 357–359. https://doi.org/10.1038/nmeth.1923

Lauro, F. M., DeMaere, M. Z., Yau, S., Brown, M. V., Ng, C., Wilkins, D., Raftery, M. J., Gibson, J. A., Andrews-Pfannkoch, C., Lewis, M., Hoffman, J. M., Thomas, T., & Cavicchioli, R. (2011). An integrative study of a meromictic lake ecosystem in Antarctica. *The ISME Journal*, *5*(5), 879–895. https://doi.org/10.1038/ismej.2010.185

Lauro, F. M., McDougald, D., Thomas, T., Williams, T. J., Egan, S., Rice, S., DeMaere, M. Z., Ting, L., Ertan, H., Johnson, J., Ferriera, S., Lapidus, A., Anderson, I., Kyrpides, N., Munk, A. C., Detter, C., Han, C. S., Brown, M. V., Robb, F. T., … Cavicchioli, R. (2009). The genomic basis of trophic strategy in marine bacteria. *Proceedings of the National Academy of Sciences of the United States of America*, *106*(37), 15527–15533.

Li, D., Liu, C.-M., Luo, R., Sadakane, K., & Lam, T.-W. (2015). MEGAHIT: An ultra-fast single-node solution for large and complex metagenomics assembly via succinct de Bruijn graph. *Bioinformatics*, *31*(10), 1674–1676. https://doi.org/10.1093/bioinformatics/btv033

Li, H., Handsaker, B., Wysoker, A., Fennell, T., Ruan, J., Homer, N., Marth, G., Abecasis, G., Durbin, R., & 1000 Genome Project Data Processing Subgroup. (2009). The Sequence Alignment/Map format and SAMtools. *Bioinformatics*, *25*(16), 2078–2079. https://doi.org/10.1093/bioinformatics/btp352

Llorens-Marès, T., Yooseph, S., Goll, J., Hoffman, J., Vila-Costa, M., Borrego, C. M., Dupont, C. L., & Casamayor, E. O. (2015). Connecting biodiversity and potential functional role in modern euxinic environments by microbial metagenomics. *The ISME Journal*, *9*(7), 1648–1661. https://doi.org/10.1038/ismej.2014.254

Lu, J., Breitwieser, F. P., Thielen, P., & Salzberg, S. L. (2017). Bracken: Estimating species abundance in metagenomics data. *PeerJ Computer Science*, *3*, e104. https://doi.org/10.7717/peerj-cs.104

Magoč, T., & Salzberg, S. L. (2011). FLASH: Fast length adjustment of short reads to improve genome assemblies. *Bioinformatics*, *27*(21), 2957–2963. https://doi.org/10.1093/bioinformatics/btr507

Mikheenko, A., Saveliev, V., & Gurevich, A. (2016). MetaQUAST: Evaluation of metagenome assemblies. *Bioinformatics*, *32*(7), 1088–1090. https://doi.org/10.1093/bioinformatics/btv697

Olm, M. R., Brown, C. T., Brooks, B., & Banfield, J. F. (2017). dRep: A tool for fast and accurate genomic comparisons that enables improved genome recovery from metagenomes through de-replication. *The ISME Journal*, *11*(12), 2864–2868.

Panwar, P., Allen, M. A., Williams, T. J., Hancock, A. M., Brazendale, S., Bevington, J., Roux, S., Páez-Espino, D., Nayfach, S., Berg, M., Schulz, F., Chen, I.-M. A., Huntemann, M., Shapiro, N., Kyrpides, N. C., Woyke, T., Eloe-Fadrosh, E. A., & Cavicchioli, R. (2020). Influence of the polar light cycle on seasonal dynamics of an Antarctic lake microbial community. *Microbiome*, *8*(1), 116. https://doi.org/10.1186/s40168-020-00889-8

Parks, D. H., Imelfort, M., Skennerton, C. T., Hugenholtz, P., & Tyson, G. W. (2015). CheckM: assessing the quality of microbial genomes recovered from isolates, single cells, and metagenomes. *Genome Research*, *25*(7), 1043–1055.

Pereira, M. B., Wallroth, M., Jonsson, V., & Kristiansson, E. (2018). Comparison of normalization methods for the analysis of metagenomic gene abundance data. *BMC Genomics*, *19*(1), 274. https://doi.org/10.1186/s12864-018-4637-6

Uritskiy, G. V., DiRuggiero, J., & Taylor, J. (2018). MetaWRAP—a flexible pipeline for genome-resolved metagenomic data analysis. *Microbiome*, *6*, 1–13.

Wei, J., Fontaine, L., Valiente, N., Dörsch, P., Hessen, D. O., & Eiler, A. (2023). Trajectories of freshwater microbial genomics and greenhouse gas saturation upon glacial retreat. *Nature Communications*, *14*(1), Article 1. https://doi.org/10.1038/s41467-023-38806-w

Wood, D. E., Lu, J., & Langmead, B. (2019). Improved metagenomic analysis with Kraken 2. *Genome Biology*, *20*(1), 257. https://doi.org/10.1186/s13059-019-1891-0

Wu, Y.-W., Simmons, B. A., & Singer, S. W. (2016). MaxBin 2.0: An automated binning algorithm to recover genomes from multiple metagenomic datasets. *Bioinformatics*, *32*(4), 605–607.

Zhu, Q., Huang, S., Gonzalez, A., McGrath, I., McDonald, D., Haiminen, N., Armstrong, G., Vázquez-Baeza, Y., Yu, J., Kuczynski, J., Sepich-Poore, G. D., Swafford, A. D., Das, P., Shaffer, J. P., Lejzerowicz, F., Belda-Ferre, P., Havulinna, A. S., Méric, G., Niiranen, T., … Knight, R. (2021). OGUs enable effective, phylogeny-aware analysis of even shallow metagenome community structures. *bioRxiv*, 2021.04.04.438427. https://doi.org/10.1101/2021.04.04.438427
